# Morphological Complexity as a Floral Signal: From Perception by Insect Pollinators to Co-Evolutionary Implications

**DOI:** 10.3390/ijms19061681

**Published:** 2018-06-06

**Authors:** Shivani Krishna, Tamar Keasar

**Affiliations:** Department of Biology and Environment, Faculty of Natural Sciences, University of Haifa, Oranim, Tivon 36006, Israel; sshivani@campus.haifa.ac.il

**Keywords:** associative learning, floral tube, perception, pollinator specialization, reward signal, symmetry

## Abstract

Morphologically complex flowers are characterized by bilateral symmetry, tube-like shapes, deep corolla tubes, fused petals, and/or poricidal anthers, all of which constrain the access of insect visitors to floral nectar and pollen rewards. Only a subset of potential pollinators, mainly large bees, learn to successfully forage on such flowers. Thus, complexity may comprise a morphological filter that restricts the range of visitors and thereby increases food intake for successful foragers. Such pollinator specialization, in turn, promotes flower constancy and reduces cross-species pollen transfer, providing fitness benefits to plants with complex flowers. Since visual signals associated with floral morphological complexity are generally honest (i.e., indicate food rewards), pollinators need to perceive and process them. Physiological studies show that bees detect distant flowers through long-wavelength sensitive photoreceptors. Bees effectively perceive complex shapes and learn the positions of contours based on their spatial frequencies. Complex flowers require long handling times by naive visitors, and become highly profitable only for experienced foragers. To explore possible pathways towards the evolution of floral complexity, we discuss cognitive mechanisms that potentially allow insects to persist on complex flowers despite low initial foraging gains, suggest experiments to test these mechanisms, and speculate on their adaptive value.

## 1. Introduction

Pollination, a major plant-insect interaction, is an important selective force that drives (combined with other forces) the evolution of flower traits. Pollinators perceive diverse floral signals and cues and modify their foraging behavior in response, thereby affecting both their own foraging success and the plants’ reproductive prospects. Here, we focus on a suite of features involved in producing morphologically complex flowers, and review their role as signals in interactions with insect pollinators. 

## 2. What Are Morphologically Complex Flowers? 

Complex flowers are structurally defined as having floral parts of many different types, which often fuse to form elaborate structures [1]. Functionally, complex flowers possess morphological features that restrict the access of insect visitors to their nectar and pollen rewards. These include bilateral symmetry (zygomorphy, see Table 1 for definitions of terms), fusion of petals, corollas that face sideways or downwards, long and/or narrow floral tubes, and concealed nectaries [2,3,4,5,6,7,8]. 

**Figure 1:**
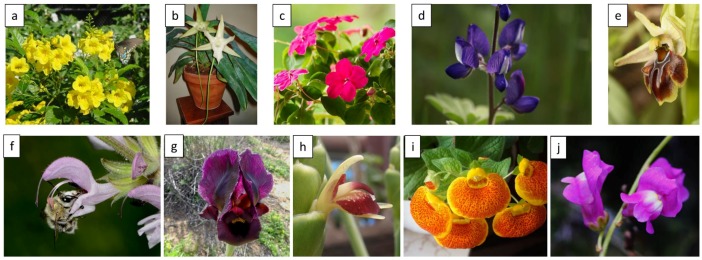
Examples of complex morphologies of flowers. (**a**) *Tecoma stans* (Bignoniaceae); (**b**) *Angaecum sesquipedale* (Orchidaceae); (**c**) *Impatiens balsamina* (Balsaminaceae); (**d**) *Lupinus* *pilosus* (Fabaceae); (**e**) *Ophrys alasiatica* (Orchidaceae); (**f**) *Salvia hierosolymitana* (Lamiaceae); (**g**) *Iris atropurpurea* (Iridaceae); (**h**) *Zingiber officinale* (Zingiberaceae); (**i**) *Calceolaria crenatiflora* (Calceolariaceae); (**j**) *Antirrhinum majus* (Plantaginaceae). Photographers: (**a**) Calvin Finch; (**b**) Karole Schon; (**d**,**i**) Judith Marcus; (**e**) Michael Pettemerides; (**f**) Gideon Pisanty; (**g**) Ada Knossow; (**h**) Alastair Culham; (**j**) George Konstantinu.

Fusion of petals, that is, sympetaly, resulted in the evolution of long tubed flowers in the lineages Ranunculales, Saxifragales, Ericales, and Dioscorales (Figure 1a) [9,10]. Some of these sympetalous flowers and a few others with polypetaly evolved further complexity in the form of spurs. These are long outgrowths that emerge from the corolla base and house nectar. Families with such spurred flowers include Tropaeolaceae, Balsaminaceae, and Orchidaceae (Figure 1b,c). A phenotypic fit between the proboscis of insects and the length of the corolla tube or spur in such flowers is critical for pollination. Apart from phenotypic fit, morphologically complex flowers, such as those with ‘keel’ flowers, require the use of mechanical force to access the nectar and pollen rewards. Such keel flowers are common in several plant families, most prominently in the Fabaceae (Figure 1d) [11,12]. Another class of complexity includes the lip forms found in Orchidaceae, Lamiaceae, Proteaceae, and Iridaceae (Figure 1e,f). Lip forms display a variety of constructions such as locked entrances in *Phlomis* and *Iris* and those with reduced lower lips in *Leonotis* where pollen is precisely deposited on the dorsal side of the pollinators [12,13,14]. Overall, zygomorphic flowers exhibit stronger floral integration compared to actinomorphic flowers, with concerted variation in structure and number of reproductive parts together with their perianth [15,16]. Table 2 summarizes the main features of floral complexity in different plant families and Figure 1 shows some examples of complex flowers.

Poricidal anthers need to be sonicated (“buzzed”) by insect visitors for the pollen to be released. Since not all pollinators are capable of buzzing [17], and since effective buzzing requires practice [18], poricidal anthers can be regarded as an additional characteristic of morphological complexity. Complex flowers often also exhibit modifications of reproductive structures, such as syncarpy (united carpels within the ovaries), increased ovule numbers, and oligostemony (≤2 whorls of stamens) [19]. Since these traits are not considered signals for pollinators, we will not discuss them further here. Morphological complexity of flowers is distinct from signal complexity, which often entails a combination of several modalities (such as color and odor) in floral advertising, and which has been discussed elsewhere [20,21].

Rewardless flowers have evolved in about 7500 species, and 85% of them belong to the family Orchidaceae [22]. While most of the orchid species are zygomorphic [23], their floral morphologies have probably been molded by diverse selection pressures. This is mainly due to the deceptive strategies involved, which range from mimicking a sympatric rewarding species to mimicry of female insects. Mimicry of complex morphologies is potentially a fine solution for enhancing trickery because visitors to the deceptive flowers cannot tell from a distance that these flowers lack reward. It has been proposed that insects visit complex deceptive flowers (and thereby select for them) as long as their associated cost in foraging efficiency is sufficiently low [24]. This idea has not been experimentally tested yet.

The structural features that make flowers complex may be directly perceived by pollinators and thus serve as visual signals or cues. Complexity could also potentially be advertised by other, correlated, floral traits that are not necessarily parts of any specific floral organ. For example, long floral tubes (a structural feature) are correlated with blue corolla color [25,26] and low achromatic contrast between corolla and background (non-structural signals) [25] in Mediterranean and temperate grassland plants. Similarly, violet corolla color correlates with the presence of a corolla tube within the crucifers of Israel [27]. The blue and violet corollas are attractive to bees [26]. In *Aquilegia*, flower traits related to complexity (length of the nectar spur and flower orientation) correlate with flower and spur color. A quantitative trait locus (QTL) analysis of these traits suggests that they are under shared genetic control [28]. An example for a non-visual and non-structural trait that indicates floral complexity is that of *Polemonium viscosum* flowers, which have two scent morphs (sweet and skunky). Each scent morph is associated with a different length of the floral tube [29]. 

## 3. How Common Are Complex Flowers?

Floral complexity is widespread, both evolutionarily and biogeographically. Buzz pollination occurs in more than 65 angiosperm families [30,31]. Zygomorphic flowers were recorded in 83 angiosperm families [5] and are known to have evolved independently from actinomorphic flowers more than 25 different times during the late cretaceous period [32,33,34,35].

As morphologically complex flowers are associated with more specialized pollination (see Section 4), increased likelihood of character displacement followed by higher diversification rates have been predicted in such taxa [35,36,37,38,39]. In line with this prediction, some of the most species-rich angiosperm families such as the Fabaceae, Lamiaceae, and Orchidaceae have complex flowers. Furthermore, sister-group analyses of zygomorphic lineages revealed higher species richness in comparison with actinomorphic lineages [36,40]. Of the 57 zygomorphic families examined by Sargent [36], 54.3% have a widespread distribution; 28% occur predominantly in Africa, Europe, and Asia; and 15.7% in the North and South Americas. However, this correlation could reflect the fact that wide geographical distribution, particularly in the tropics, also promotes speciation and diversification of plants. Characteristics such as herbaceous habit, hermaphroditism, and absence of fleshy fruits were also associated (albeit weakly) with zygomorphy. These traits may also act in concert with floral complexity to promote speciation [5,40].

## 4. The Role of Morphological Complexity in Specialization of Plant–Pollinator Interactions

It has been repeatedly hypothesized that only a subset of all pollinators are capable of reaching the nectar and pollen rewards in morphologically complex flowers, whereas simple flowers are accessible to a wide range of visitor species. This was predicted to reduce the number of pollinator species that forage on complex flowers [41], and potentially also visitation rates [42]. The evidence for these predictions from observational studies is mixed. The length and width of the nectar tube were found to act as filters for nectar foragers, by barring visitors whose proboscises are shorter or wider than the tube. Thus, flowers with long and narrow corolla tubes were visited by fewer pollinator species than short-tube flowers [43]. Similarly, simple flowers were visited by a more diverse insect assemblage than complex flowers in four Himalayan mountain plant communities [41]. In European alpine communities, higher local foraging specialization of bees (i.e., fewer plant species visited at an observation site) correlated with high frequency of visits to long-tubed flowers. However, this correlation was weaker in flies and non-existent in Lepidoptera [44], possibly because their flexible proboscis allows access to flowers of many shapes. In addition, a study that classified flowers of 1403 plant species as either “open” or “closed”, based on corolla morphology, did not find a wider range of visitors to open flowers than to closed ones [45]. Similarly, the number of visitor species was not affected by floral morphology in species-rich pollination networks in the Peruvian Andes [46].

Similar conflicting findings concern the relationship between floral complexity and visitation rates. As predicted, tubular flowers received fewer visits than open ones in plant communities from several floristic regions [42,47]. Long-tubed artificial flowers received fewer bumblebee visits than short-tubed flowers in laboratory experiments [7,48]. Two other studies, on the other hand, found different trends: in a Norwegian grassland, actinomorphic open flowers received more visits by flies than zygomorphic closed flowers, whereas visit rates by bumblebees were not influenced by flower morphology [49]. *Salvia nipponica* flowers, which were manipulated in the field for reduced complexity, received visits by bumblebees at the same rate as intact flowers [50].

In situations where complex flowers attract fewer pollinator species and receive fewer visits than simple flowers, their abundance and spatial distribution may be affected. As a test of this hypothesis, morphological complexity of flowers within 427 plant species from the Greek flora was examined in the context of vulnerability of extinction risk. Complex morphological shapes such as tube, flag, gullet, and trap with bilateral symmetries were found to be relatively more vulnerable compared to the open shapes with radial symmetry. This could be due to insufficient pollination experienced by plants with complex flowers [8]. In agreement with this interpretation, high-elevation flower communities in the Switzerland and Italy (where pollinator diversity is low and bees are scarce) have mostly simple morphologies. Lower-altitude communities with high bee abundance have more diverse morphologies and higher proportions of complex flowers [51,52]. 

## 5. Benefits of Complex Flower Shapes: The Plant Perspective

What selective advantages maintain complex flowers in plant communities, even though they attract fewer pollinators? It has been proposed that insect species that can handle complex flowers are also highly effective pollinators, and thus increase the plants’ pollination success. In particular, the “filtering” of pollinators by their ability to handle floral complexity was hypothesized to enhance pollinator constancy. Highly specialized pollinators were proposed to mainly exploit complex flowers of a few species, rather than shifting among the many species with simple morphologies. This could reduce the costs of inter-specific pollen transfer, providing a selective benefit to floral complexity for the plants [53]. 

Tests of the effects of floral complexity on flower constancy yielded equivocal results. In one field study, plants with complex flowers shared fewer insect visitors with a simple-flowered focal plant (*Euphorbia esula*), compared with other simple-flowered species in the same plant community. In addition, fewer pollen grains of the focal species were deposited on the stigmas of complex flowers as compared to simple flowers, confirming higher conspecific pollen transfer in species with complex flowers [54]. Other studies, however, did not show the predicted pattern: in one of them, the cumulative (season-long) deposition of heterospecific pollen was higher on the stigmas of zygomorphic flowers than on actinomorphic ones [55]. In another, there was no effect of floral symmetry on the frequency of pollen transfer among 19 plant species across three plant communities [56]. A laboratory study characterized flower constancy by recording the visit sequences of bumblebees on floral arrays that contained pairwise combinations of four flower species. Two of the species were morphologically complex and the remaining two were simple. The bees were most constant to one of the complex species, but least constant to the other [57]. 

A related putative benefit of complex flowers for plants is a reduced risk of geitonogamous selfing. As floral handling times are increased, the relative costs of travel time diminish in terms of foraging efficiency. This may induce visitors to make longer flights, resulting in fewer successive visits per plant. Bumblebees that foraged on manipulated *Salvia nipponica* flowers, in which the access to the nectaries was experimentally simplified, indeed shifted less often among plants than bees that visited intact flowers [50].

Bilateral symmetry was also suggested to promote more consistent entry by pollinators into the flower. This could increase the contact of floral sex organs with the insect’s body and improve the plant’s reproductive prospects. The angle of entry by bumblebees into dissymmetric artificial flowers was in fact more consistent than when they entered radially symmetric flowers [58]. 

## 6. Benefits of Complex Flower Shapes: The Pollinator Perspective. Is Floral Complexity an Honest Signal of Reward?

From the pollinators’ point of view, floral complexity has been suggested to signal high foraging rewards. The rationale for this expectation is twofold: at the evolutionary level, since complex flowers can be pollinated by only a few specialized insects, they would be selected for higher nectar and pollen production rates [59,60]. At the proximate level, complex flowers receive fewer foraging visits, and thus are expected to have higher standing crops of food rewards because of reduced consumption by insects. Consistent with these predictions, nectar production rates correlated positively with flower tube depth across eight species of Ericaceae [61], among hundreds of hummingbird-pollinated plants [62,63], and among 76 Mediterranean bee-pollinated phrygana plants [64]. 

High-quality pollen is an additional reward that is crucial for insect pollinators and is offered by complex flowers. The quality of pollen sources is determined by their amino acid constitution, protein content, and mineral composition [65]. Pollen varies from as low as 2.5% to 61% in the content of protein dry mass [66]. Size, longevity, and reproductive output of pollinators [67,68,69], as well as the health of entire bee colonies, depend on the quality and quantity of pollen received [70,71,72,73]. Bees are known to assess pollen amounts and properties and prefer pollen-rich flowers [74,75,76,77]. 

It has been hypothesized that complex pollen-rewarding and buzz-pollinated flowers produce better-quality pollen that attracts their pollinators [78,79]. In a study with 377 plant species from 93 families, buzz-pollinated clades contained pollen grains rich in protein content compared to the other clades [79]. A study that examined foraging preferences and diet breadth of bumblebees in the southern UK found that they preferred pollen of Fabaceae members compared to that of simpler inflorescences of Asteraceae [80]. It is likely that pollen of Fabaceae species is richer in its protein content compared to Asteraceae pollen, due to their symbiosis with nitrogen-fixing bacteria [81,82].

## 7. Perception and Processing of Floral Complexity Signals 

Detection and discrimination of flowers is of critical importance for insects that depend on their food (and various other) rewards. Pollinators combine odor and visual information to accomplish this task. While we know various aspects of insect visual systems in great detail, particularly that of honeybees, it is still unclear how they perceive and integrate combinations of signals such as color, intensity, size, shape, pattern, and symmetry advertised by flowers. As morphologically complex flowers possess these myriad visual features, we do not yet fully understand how insects perceive these flowers. Below, we introduce key features of the visual system of bees as a well-studied model, and discuss some of the aspects pertaining to sensory perception of patterns and symmetry associated with complex flower shapes.

Most insects have compound eyes with a mosaic of optical units (ommatidia). Each ommatidium contains photoreceptor cells with photopigments, which capture the incoming photons. Photoreceptors are typically G-protein coupled receptors with an opsin protein and a chromophore, of which retinal is the most common. Light is absorbed by the chromophore, which in turn isomerizes, causing conformational change in the opsin. This results in a phototransduction cascade (see [83] and references therein for a general introduction). Insects exhibit a diverse array of photoreceptor cells, varying from three in honeybees to eight in *Papilio* spp. Photoreceptors that are sensitive in the long-wavelength region (“green” receptors) are the most common type in insect taxa that possess four receptors or more [84,85,86]. 

Bees typically possess a trichromatic visual system with photoreceptors that are sensitive in the UV, blue, and green regions [87]. Chromatic vision, required for perceiving color signals, is mainly achieved by neuronal comparison of signals (color-opponency) from all the three receptor types [88]. Achromatic vision, needed for detecting floral shapes, involves the long wavelength receptor alone [89,90].

To extract information from visual features of flowers, insects utilize achromatic as well as chromatic information. The detection itself depends on the size and distance of the flower from the insect [89]. Typically, the insects need to be quite close to the flowers (20–30 cm), to recognize and resolve their visual features [91]. Characteristics of the flowers such as color, shape, and size, along with the insect’s structure of the eye, inter-ommatidial angle, and angular size of photoreceptor fields affect visual acuity [92].

Depending on the visual system of the insect, the flower needs to be projected on its eye in a way that a critical number of ommatidia receive a sufficient signal for excitation. This number of ommatidia depends on the intensity of the contrast provided by the flower against the background. For flowers without such green contrast (such as flowers within dense inflorescences), the number of ommatidia required is much larger [89]. Therefore, understanding how color contrast to the background interacts with detection of patterns presented by flowers is significant. Honeybees trained to a bilaterally symmetrical flower of three different colors, and tested with different angular orientations of the same, performed better with decreasing color distance to the background (perceptual difference). To explain this finding, a hierarchical system was suggested, where recognition of color precedes pattern recognition. Consequently, with increasing color contrast to the background, the processing of floral color takes precedence and the accuracy to perceive patterns declines [93]. 

After perceiving floral shapes, pollinators associate them with food rewards. Behavioral experiments indicated an innate preference for bilateral symmetry in honeybees [94]. However, a later study suggested that these preferences could have resulted from the pre-training procedure used in the experiment [95]. Early work showed that honeybees trained to relatively simple radial models preferred complex disruptive patterns in choice tests. When trained to complex patterns, they showed increased response to such patterns. In later experiments, honeybees were trained to discriminate bilaterally symmetric from non-symmetric patterns, and also transferred this discrimination to novel patterns [96,97]. Bumblebees preferentially visited cone-like and tubular shapes compared to flat models [98,99,100], while in honeybees no such significant preference for depth was recorded [101]. These findings confirm earlier field observations that zygomorphic flowers were visited relatively more by bumblebees than by honeybees [102].

These behavioral studies indicate that elements of floral complexity, such as bilateral symmetry, are easily recognized and learned by insect pollinators. Features such as shape and patterns have been debated to be recognized using either retinotopic snapshots (template hypothesis [93]) or by extracting important image features (pattern hypothesis [103]). Some features known to be extracted by bees include contour lengths, their densities, and the area occupied by pattern [104]. Currently, a conceptual framework that integrates both hypotheses is widely accepted, where extracted features are combined into an ordered alignment for recognizing flowers [105,106]. Additionally, symmetry detectors in the neuronal systems of flower-visiting insects have been suggested (e.g., [96,103,104,107]). As flowers with different symmetries vary in their contrasts of the boundaries, mechanisms used to detect radial symmetry might differ from those used to identify bilateral symmetry [103]. Based on behavioral experiments in *Apis mellifera*, Srinivasan and colleagues suggest the use of geometrical cues to differentiate orientation of patterns such as stripes and checkerboards [107]. This was proposed to indicate the presence of three channels that are sensitive to different orientations and possibly separated by 120 degrees from each other, due to the ommatidial hexagonal arrangement. Cells that function to detect lines and edges are also known to be present in insects, and help to detect global and local features of objects [108,109].

The importance of the position of objects within the visual field for pattern recognition has been demonstrated in various insects [110,111,112]. By training bees to varying inclinations of black and white contrasting pattern, the lower part of the frontal visual field was elegantly demonstrated as crucial for pattern recognition [112]. This also endorses the presence of “space-constant fibres” in the optic ganglia of bee eyes, which are sensitive to spatial directions [113]. 

In conclusion, features of floral complexity are perceived by the visual system of insects, based mostly on excitation of long-wavelength color receptors in the lower part of the field of vision. Bees readily learn to associate these features with food rewards. Several neural mechanisms were suggested to underlie the learning process, but have not yet been experimentally confirmed.

## 8. Learning to Handle Complex Flowers

Flowers with complex morphologies are learned more slowly than simple flowers [114]. Handling and travel times increase when bees forage on more than one type of flower at the same time, especially if flowers are complex [4,115,116,117]. Yet once learned, flower-handling techniques are retained in memory for days or even weeks [118,119]. Thus, new complex flower types are initially less profitable to foraging bees than familiar or simple flowers with the same reward. Nevertheless, new complex flowers become more profitable if bees persist and learn how to handle them, resulting in greatly reduced handling times and higher food intake rates. This is because a much larger improvement in handling proficiency is expected for complex flowers than for simple ones [114]. 

Energy intake rate (calories ingested/time) is a popular currency for evaluating foraging efficiency [120]. Alternatively, the ratio between energy intake and expenditure can provide a measure of foraging success [121]. The handling of new, complex flowers by pollinators is inefficient by both currencies, since it requires more time and energy than the handling of simple sources. From a foraging perspective, this raises questions about the cognitive and behavioral mechanisms that cause animals to persevere on complex food sources until they have learned to handle them effectively. 

In an ongoing research project, we are exploring mechanisms that may allow bumblebees to learn complex tasks in spite of initially low rewards in the presence of alternatives. We are considering the following hypotheses to explain how bumblebees may accept complex flowers with high future but low immediate rewards, even in competition with simple flowers with moderate amounts of immediate rewards:

**Hypothesis** **1.** *Bumblebees may use their speed of improvement in reward intake, in addition to the mean recent intake rates, to anticipate the future profitability of complex flowers. In other words, rapid improvement in the handling of complex flowers may reinforce the bees’ attempts to visit such flowers. This strategy could cause bees to persist on resources that require long handling times initially, but that are highly profitable to experienced foragers. During initial learning, the low intake rate from these flowers may be compensated by the high improvement rate, and bees could rate such flowers as high-potential. After bees learn to handle the complex flowers, the reward intake rate would increase, while the improvement rate would decrease.*

**Hypothesis** **2.** *Bumblebees may use the amount or quality of food collected, separately from the costs of handling a flower, to evaluate the potential profitability of morphologically complex flowers. Handling times may enter into flower choice independently. Thus, inexperienced bees could evaluate high-reward complex flowers as low on intake rates, but high on reward amount. This provides an indication that potential reward rates are high if handling time can be shortened. Experienced foragers would score such flowers higher on recent intake rates, while their evaluation in terms of reward volume would remain unchanged. Choices of honeybees between food sources that differ both in rewards and handling times are consistent with a separate evaluation of each parameter* [122,123]*.*

**Hypothesis** **3.** *Once a bee has experienced a higher reward rate on a complex flower, this may prime a preference for complex flowers generally in future encounters. Individual sampling behavior may be of sufficient frequency that some bees will discover how to handle complex flowers. If experience can create a bias towards complex morphology, this may enable some bees to exploit additional rewarding complex flowers in their habitat.*

Experiments to test these hypotheses combine exposure of bumblebees to real and artificial flowers of varying morphological complexity. We aim to manipulate, independently, the flowers’ initial and steady-state handling times, their rewards, and the bees’ previous foraging experience, to assess whether and how these putative mechanisms interact to explain learning of complex morphologies.

## 9. Conclusions and Future Directions 

Floral morphological complexity is an important dimension for interpreting the evolution of plant–pollinator interactions. To understand the persistence and spread of floral complexity in angiosperms, the sensory signals associated with complex morphologies should be identified. Complex structures appear to occur together with other floral signals and cues to enhance the attraction of complex flowers to pollinators, and function as a sieve favoring interacting insects with specific traits that aid pollination efficiency. Of the many hypotheses that have been put forward to explain the evolution of complexity, efficient pollen receipt has received considerable support. Recent molecular advances in our understanding of flower-shape evolution and of insect sensory capabilities provide clues as to the origins of complexity. Ongoing work investigates potential learning mechanisms that could induce pollinators to visit complex flowers despite their low initial foraging profitability. From a sensory physiology perspective, the coding of the behavioral repertoire necessary for extracting rewards from complex flowers is a significant aspect to unravel. We propose that developing quantitative measures to score and compare floral complexity among species (see [6,8] for promising approaches), is an additional important direction for future research.

## Figures and Tables

**Table 1:** Glossary of terms used in this review.

Term	Definition
Achromatic stimuli	Visual stimuli that vary only in the total intensity of reflected light.
Actinomorphy	Two or more planes of symmetry; radial symmetry.
Chromatic stimuli	Visual stimuli that vary only in the spectral (wavelength) composition of the reflected light (color).
Color distance	A metric indicative of perceptual color difference between two stimuli in animal color spaces (graphical models based on photoreceptor properties and sensitivities).
Color-opponency	Combination of differential neuronal outputs of color-sensitive photoreceptors to create a signal in the processing of color.
Floral integration	Covariation in flower parts.
Flower constancy	Tendency for an individual pollinator to visit flowers of a single species within a foraging bout.
Geitonogamy	A type of self-pollination, in which a flower is fertilized by pollen from another flower of the same individual plant.
Nectary	Specialized cells that are usually part of a flower, which secrete sugary fluids.
Photopigment	A chemical that undergoes a chemical change when exposed to light. In vision, these are primarily the visual pigments or other opsin-based molecules.
Poricidal anthers	Anthers packed with loose pollen grains, dehiscing by a pore at one end of the thecae.
Trichromacy	A color-vision system based on three classes of color receptors.
Visual acuity	The minimum angular separation between two objects in the visual field that are perceived as distinct, at a given distance from the viewer.
Visual field	The limits of the space around the eyes from which visual information is obtained.
Zygomorphy	A single plane of symmetry; bilateral symmetry; one half of an object mirrors the other half.

**Table 2:** Features of morphological complexity in flowers of selected plant families.

Family	Floral Morphology	Pollinators	Examples (Genera)
Acanthaceae	Fused corolla lobes, usually bilabiate (upper lip suppressed and larger lower lip in some species)	Bees, hummingbirds, flies, moths	*Acanthus*, *Justicia*
Balsaminaceae	Four petals combined in pairs and one upper petal, usually 3–5 sepals, one of which forms a long tube called spur	Bees	*Impatiens*, *Hydrocera*
Bignoniaceae	Fused corolla lobes (usually five), bilabiate, large and showy, with wider upper part	Bees, bats, hummingbirds	*Tecoma*, *Incarvillea*, *Spathodea*
Boraginaceae	Five petals fused, sometimes lobed, forming a tube or funnel shape with infoldings or scales	Bees, butterflies, hummingbirds	*Onosma*, *Heliotropium*
Cannaceae	Corolla three-lobed, forming a tube together with stamen and staminodes	Bees, birds	*Canna*
Caprifoliaceae	Five fused corolla lobes forming a tube or funnel shape	Bees, butterflies, moths, hummingbirds	*Lonicera*, *Abelia*
Fabaceae	Flag is formed by single median petal and the keel is composed of two petals (in lower lateral position) which secondarily join into a common boat-shaped petal	Bees	*Lupinus*, *Lotus*
Goodeniaceae	Five unified corolla lobes either uni-or bilabiate, stamens form a tube-like structure	Bees	*Dampiera*, *Scaevola*
Iridaceae	Corolla is formed by three inner and three outer segments, free or united	Bees, birds	*Iris*, *Crocosmia*
Lamiaceae	4–5 corolla lobes often reduced to 2–3, with two lips. Upper lip is two-lobed and lower lip is three-lobed. Lower lip occasionally hooded or concave	Bees, hummingbirds, flies	*Salvia*, *Plectranthus*, *Lamium*
Moringaceae	Five petals, unequal and overlapping; petaloid sepal; resemble inverted keel flowers of Fabaceae	Bees	*Moringa*
Orchidaceae	Three petaloid sepals and three petals, variable in shape and color, sometimes spurred or with enlarged sac-like tepal. The inner median, anterior tepal is enlarged and is called the labellum	Bees	*Ophrys*, *Catasetum*
Proteaceae	Four slender petaloid sepals, distinct or united, forming a tubular structure, petals usually absent	Birds, beetles	*Grevillea*, *Conospermum*
Resedaceae	Clawed petals, fringed, bifid, vary in number from 2 to 8, innermost petal is large and outer ones are smaller	Bees	*Reseda*
Scrophulariaceae	Bell-shaped corollas with variations including narrow corolla tube, spurs, keel petal	Bees, hummingbirds	*Digitalis*, *Linaria*, *Antirrhinum*
Valerianaceae	Five overlapping corolla lobes, sometimes fused, basal nectar-filled spur	Butterflies	*Centranthus*
Vochysiaceae	Five overlapping corolla lobes, fused, basal nectar filled spur	Bees, butterflies	*Vochysia, Callisthene*
Zingiberaceae	Tubular corolla with three lobes, colored petaloid labellum derived from staminodes	Bees	*Mantisia*, *Zingiber*
